# Mediterranean Diet Adherence and Cardiometabolic, Inflammatory, Hormonal, Body Composition, and Behavioral Health Profiles in Physically Active Young Adults: An Analytical Cross-Sectional Study

**DOI:** 10.3390/healthcare14193345

**Published:** 2026-10-08

**Authors:** Nour Amin Elsahoryi, Huda Awni Al-Ramahi, Omar Amin Alhaj, Haitham Jahrami

**Affiliations:** 1 Department of Nutrition, Faculty of Pharmacy and Medical Sciences, University of Petra, Amman 11196, Jordan; 202311090@uopstd.edu.jo (H.A.A.-R.); omar.alhaj@uop.edu.jo (O.A.A.); 2 Government Hospitals, Manama 329, Bahrain; hjahrami@hospitals.gov.bh; 3 Department of Psychiatry, College of Medicine and Health Sciences, Arabian Gulf University, Manama 329, Bahrain

**Keywords:** appetite-related hormones, biomarkers, cross-sectional study, Jordan, Mediterranean diet, physically active young adults, cardiometabolic health

## Abstract

**Background:** The Mediterranean Diet (MD) is linked to better cardiometabolic and inflammatory health, but its relationships with appetite-related hormones, body composition, and behavioral health remain unclear in physically active young adults from understudied Arab settings. Objective: To examine associations between MD adherence and cardiometabolic, inflammatory, hormonal, body-composition, and behavioral-health profiles, including sleep quality, physical activity, perceived recovery, and health-related quality of life (HRQoL), among physically active young adults in Jordan. **Methods:** Eighty adults aged 18–35 years (44 men and 36 women) were recruited from fitness centers and campuses in Amman. MD adherence was assessed using the validated Moroccan Arabic version of the 14-item Mediterranean Diet Adherence Screener (MEDAS; adherent ≥ 8). Fasting lipid profile, C-reactive protein (CRP), interleukin-6 (IL-6), leptin, and ghrelin were measured using enzymatic and ELISA methods. Anthropometric and body-composition measures were assessed using stadiometry and multifrequency bioelectrical impedance analysis. Sleep quality, physical activity, perceived recovery, and HRQoL were assessed using validated questionnaires. **Results:** 60% of participants were non-adherent to the MD. In exploratory analyses, non-adherence was associated with higher odds of abnormal triglycerides (adjusted OR = 9.17, 95% CI: 2.08–40.38). After additional adjustment for percent body fat, the association with abnormal CRP remained significant (adjusted OR = 5.39, 95% CI: 1.34–21.61), whereas the association with abnormal leptin was attenuated and no longer statistically significant (adjusted OR = 3.47, 95% CI: 0.75–16.07). The leptin finding was sensitive to adjustment for adiposity, while residual confounding remains possible for the CRP association. Non-adherent participants had higher BMI, body fat mass, and percent body fat, and lower physical HRQoL. No statistically significant associations were detected for ghrelin, resting blood pressure, or resting heart rate. The IL-6 findings were inconclusive because 62.5% of measurements were below the assay detection limit. **Conclusions:** Greater MD adherence was associated with a more favorable cardiometabolic and body-composition profile in this exploratory cross-sectional sample. These hypothesis-generating findings highlight the importance of considering dietary quality alongside physical activity and require confirmation in larger prospective studies with more comprehensive confounder adjustment.

## 1. Introduction

The Mediterranean diet (MD) is a traditional eating pattern that involves high consumption of plant-based foods, such as vegetables, fruits, whole grains, legumes, nuts and seeds, and extra virgin olive oil as a key component of dietary fat; moderate intake of fish, seafood and fermented dairy; and limited intake of red meat and processed meats [1]. It has been extensively investigated in relation to cardiovascular and metabolic outcomes, systemic inflammatory markers, and longevity [2]. Healthy dietary patterns may be particularly relevant in physically active populations because they support metabolic health, exercise recovery, inflammatory regulation, body composition, and functional capacity [3]. Observational evidence links greater Mediterranean diet adherence with more favorable metabolic profiles [4], while randomized dietary-pattern evidence supports potential effects on selected inflammatory biomarkers [5]. However, these findings derive from heterogeneous populations and do not specifically establish these associations among non-elite physically active young adults. Most studies have involved elite athletes or disease-specific populations, with the number of young adults who are physically active in settings outside professional sports remaining relatively understudied [6]. Evidence concerning appetite-regulating hormones also remains limited. Leptin, which is secreted by adipose tissue in near-direct proportion to fat mass, and ghrelin, the principal orexigenic hormone produced in the stomach, are key regulators of energy balance and metabolic homeostasis, and disruption of their levels is associated with adiposity, insulin resistance, and systemic inflammation [7]. Their relationships with adherence to MD among physically active young adults, however, have been sparsely investigated in empirical literature, especially in the context of objective and quantitative measurement via ELISA. Most of the available literature has relied on self-reported dietary assessment without incorporating biological endpoints [1]. Likewise, the behavioral aspect of this question, which covers sleep quality, perceived recovery, activity level and health-related quality of life (HRQoL), has been studied little in this context. Some of the outcomes measured in PSQI, IPAQ-SF, PRS and SF-12 have not been investigated together with dietary data and biomarker data simultaneously in previous studies [8,9]. Jordan remains underrepresented in this field of research. Although Jordan lies within the Eastern Mediterranean region, the country is undergoing a nutritional transition toward energy-dense and highly processed dietary patterns, particularly among younger adults [10,11,12]. Previous research has reported suboptimal physical-activity levels and sex-related differences in physical-activity patterns among Jordanian university students [13]. This study, to the best of our knowledge, is one of the first to assess adherence to the Mediterranean diet in relation to metabolic, inflammatory and appetite-related biomarkers, multifrequency bioelectrical-impedance measurements, and validated questionnaires related to behavioral-health components in physically active young adults.

This study was designed to address these converging gaps. By integrating objective laboratory measurements with validated behavioral instruments, this study aimed to examine associations between MD adherence and cardiometabolic, inflammatory, hormonal, body-composition, and behavioral-health profiles among physically active young adults in Jordan.

## 2. Materials and Methods

### 2.1. Study Design and Participant Recruitment

This analytical cross-sectional study was conducted in compliance with the Declaration of Helsinki and reported in accordance with the STROBE guidelines for observational studies [14]. The research ethics committee of the Faculty of Pharmacy and Medical Sciences, University of Petra, gave ethical approval (Approval No. S/13/8/2025, decision date 17 August 2025). Participants were recruited through a non-probability purposive sampling strategy using study flyers distributed across five university campuses and seven fitness centers/sports clubs in Amman, Jordan. Individuals who responded to the advertisement were screened against the prespecified eligibility criteria.

Adults aged 18–35 years who reported performing at least 150 min of moderate-intensity physical activity per week, consistent with WHO guidelines [15], were eligible to participate. All elite or professional athletes, individuals with any diagnosis of chronic disease (diabetes, cardiovascular disease, or metabolic disorders), pregnant or lactating women, and those taking dietary supplements or medications that would likely impact the biomarkers of interest were excluded. Participants attended two study visits. On the first visit, written informed consent was obtained, questionnaires were administered, and body-composition measurements were performed. Fasting blood samples and clinical measurements were obtained during a second morning visit. The sociodemographic data collected included gender, age, marital status, current education level, smoking status, and monthly personal income. Education level reflected the participant’s current degree level and was categorized as undergraduate (bachelor’s degree) or postgraduate (master’s or doctoral degree). Personal monthly income was self-reported and categorized as <300, 300–500, or >500 Jordanian dinars (JD). These cut-points were selected to align with income categories used in a national Jordanian survey [16]. Sample size was estimated a priori using G*Power (v.3.1.9.7) based on binary logistic regression, with α = 0.05, power = 0.95, and a medium effect size (Nagelkerke R^2^ = 0.15), yielding a minimum of 77 participants [17]. Eighty participants were recruited to allow for incomplete data. Given the exploratory nature of the study and the number of outcomes examined, all regression findings are treated as exploratory and interpreted accordingly.

### 2.2. Mediterranean Diet Assessment

MD adherence was measured using the validated Moroccan Arabic version of the 14-item MEDAS, translated and validated by Sammoud et al. (2023) [18]. The MEDAS comprises 12 food-frequency questions and two dietary habit questions, each scored 0 or 1, yielding a total score of 0–14. A validated cut-off of ≥8 was used to classify participants as adherent or non-adherent for clinically interpretable group comparisons, based on the ROC-derived threshold reported in the Moroccan Arabic validation study (sensitivity = 0.81, specificity = 0.57) [18]. To avoid relying solely on dichotomization and to preserve the ordered information contained in the 0–14 score, MEDAS was also analyzed as a continuous variable in the correlation analyses. The Moroccan Arabic version demonstrated strong psychometric properties, including excellent internal consistency (KR-21 = 0.851) and test–retest reliability (ICC = 0.876, 95% CI: 0.831–0.909) [18]. It should be noted that this instrument was validated in a Moroccan Arabic-speaking population; the absence of a Jordan-specific validation is acknowledged as a limitation of the present study.

### 2.3. Biomarker Assessment

Participants were instructed to observe a minimum 12 h fast and abstain from structured exercise for ≥24 h prior to venipuncture. Fasting serum TG was measured directly using the HP-AFS/1-PLUS Analyzer (HIPRO) by a validated enzymatic colorimetric method. Total cholesterol (TC) and HDL-C were also measured on the same analyzer by validated enzymatic colorimetric methods, and LDL-C was calculated from TC, HDL-C, and TG. During data verification, HDL-C showed an implausible distribution: 41 of 77 valid measurements (53.2%) were recorded at exactly 3.55 mmol/L despite a manufacturer-specified validated linearity range of 0.10–3.90 mmol/L for this assay and the absence of any differential dilution or re-analysis across samples, and three further values fell below the assay’s lower analytical limit. No technical explanation for this pattern could be identified. Because TC was measured on the same platform and in the same analytical run as HDL-C, and LDL-C was calculated from TC, HDL-C, and TG, the reliability of all three analytes could not be established; TC, HDL-C, and LDL-C were therefore not analyzed (see Section Strengths and Limitations). CRP was quantified by chemiluminescence immunoassay on the same platform; three values exceeding the upper limit of quantification (10.0 mg/L) were top-coded at that boundary prior to analysis [19]. Serum IL-6 (pg/mL), leptin (ng/mL), and ghrelin (pg/mL) were quantified using sandwich ELISA kits (FineTest^®^ EH0201, EH0216, and EH0355, respectively) at 450 nm absorbance. Raw optical density readings were blank-corrected and converted to concentrations using kit-specific standard curves fitted by four-parameter logistic (4PL) regression, in accordance with the manufacturer’s protocol (FineTest EH0201, EH0216, EH0355); samples were multiplied by the respective dilution factors (2× for IL-6 and ghrelin; 10× for leptin). Values below the assay limit of detection (LOD) were substituted at LOD/2 prior to analysis (IL-6: LOD = 2.813 pg/mL, 62.5% censored; ghrelin: 1.125 pg/mL, 6.25% censored; leptin: 0.1875 ng/mL, 2.5% censored) [19,20]. The high IL-6 censoring rate is biologically consistent with the low resting concentrations expected in healthy, non-obese, physically active young adults and constitutes a methodological limitation that renders IL-6 group comparisons exploratory [21]. Capillary fasting blood glucose (FBG) was measured using the Procheck Advance Multi-Functional Monitoring System (TaiDoc, New Taipei City, Taiwan) following a minimum 8 h overnight fast and was screened against American Diabetes Association glycemic thresholds for indicative classification only, recognizing that capillary whole-blood measurements are not equivalent to venous plasma glucose and should not be used for formal diagnosis [22]. Resting blood pressure and resting heart rate (RHR) were measured in triplicate using an Omron M7 Intelli IT sphygmomanometer under standardized seated conditions.

### 2.4. Anthropometric and Body Composition Assessment

Standing height was measured using a stadiometer. Body weight was measured using the InBody 970 multifrequency bioelectrical impedance analyzer (InBody Co., Seoul, Republic of Korea). Height and gender were entered into the device, which automatically calculated BMI and generated the body-composition outputs. BMI was treated as an anthropometric indicator, whereas body fat mass (BFM), percent body fat (PBF), fat-free mass (FFM), total body water (TBW), skeletal muscle mass (SMM), and skeletal muscle index (SMI) were treated as body-composition indicators. Measurements were conducted after a minimum 4 h fast, with participants in standardized light clothing and free of metallic accessories. Female participants were assessed without standardization for menstrual-cycle phase, which was not systematically documented in this study.

### 2.5. Behavioral Assessments

Physical activity was assessed using the validated Arabic version of the International Physical Activity Questionnaire–Short Form (IPAQ-SF), a self-reported measure; no objective activity-monitoring device was used.

Weekly MET-minutes were calculated using standard coefficients for walking, moderate-intensity activity, and vigorous-intensity activity of 3.3, 4.0, and 8.0 METs, respectively. Participants were classified as moderately or highly active according to standard IPAQ scoring criteria [23]. The validated Arabic version of the Pittsburgh Sleep Quality Index (PSQI) was used to assess sleep quality, with a global score >5 indicating poor sleep quality [24]. Perceived recovery was assessed using the single-item 0–10 Perceived Recovery Status scale [25]. For the present analysis, scores were categorized using prespecified study-defined cutoffs as poor (0–4), moderate (5–7), or excellent (8–10). These categories were defined by the study team for descriptive purposes and have not been independently validated; the original PRS scale is designed to be analyzed as a continuous measure [25]. The PRS was translated into Arabic using forward and backward translation and reviewed for linguistic and conceptual equivalence. In a preliminary validation assessment conducted by the research team in 30 participants, the Arabic version demonstrated excellent test–retest reliability over a 10-day interval (ICC = 0.989, 95% CI: 0.977–0.995). HRQoL was measured by the validated Arabic SF-12 Health Survey [26]. Physical-oriented and mental-oriented HRQoL scores were calculated from the relevant item responses and transformed to percentage scores ranging from 0 to 100, with higher scores indicating better health status.

### 2.6. Dietary Intake Assessment

Three-day dietary records, including two weekdays and one weekend day, were collected to characterize macronutrient and micronutrient intake across adherence groups. ESHA Food Processor (v.8.6) was used to analyze the data. The dietary records were used to provide complementary information on energy and nutrient intake and were not used to determine Mediterranean diet adherence. The adherence group-specific data for all macronutrients and micronutrients are provided in Supplementary Table S1. Given the exploratory design, no single confirmatory primary outcome was designated. The health outcome domains examined in relation to MD adherence comprised cardiometabolic and physiological indicators, inflammatory biomarkers, appetite-related hormones, anthropometric and body-composition measures, and behavioral-health and HRQoL outcomes.

### 2.7. Statistical Analysis

All data were analyzed using IBM SPSS Statistics (v.28.0). Continuous variables are presented as median (IQR), and categorical variables as n (%). Unadjusted exploratory comparisons of sociodemographic and behavioral distributions across MEDAS adherence groups were performed using Pearson’s chi-square test or Fisher’s exact test, as appropriate, with Phi (φ) and Cramér’s V reported as effect-size measures. These analyses were used solely to characterize the sample and were not used to infer independent associations or to select covariates for the regression models. Continuous biomarker, body-composition, and HRQoL outcomes were compared between adherence groups using the Mann–Whitney U test, while associations between the continuous MEDAS score and selected continuous health outcomes were assessed using Spearman’s rank correlation. Binary logistic regression was used as a secondary exploratory analysis to estimate age- and gender-adjusted odds ratios for abnormal clinical outcomes associated with MEDAS non-adherence. Given the modest sample size and sparse outcome events, parsimonious hierarchical models were used: Block 1 included age and gender, and Block 2 added the MEDAS adherence group. Age was entered as a continuous variable. Gender was entered as a categorical variable with male as the reference category, and MEDAS adherence was entered as a categorical variable with the adherent group as the reference category. For CRP and leptin, additional sensitivity models further adjusted for percent body fat (PBF) to evaluate the influence of adiposity on the observed associations; these sensitivity estimates are reported in Section 3. Accordingly, the regression estimates were interpreted as minimally adjusted, hypothesis-generating associations rather than as independent or causal effects. Model fit was evaluated by the Nagelkerke R^2^ and Hosmer–Lemeshow test. All tests were two-tailed, and *p* < 0.05 was treated as a nominal significance threshold. Because the study was exploratory and hypothesis-generating rather than confirmatory, no formal multiplicity correction was applied. Nominal *p*-values were therefore interpreted cautiously alongside effect sizes, confidence intervals, biological plausibility, and consistency across analyses, and no individual finding was treated as confirmatory evidence. The reference range used to define the abnormal TG category was that provided in the reagent manufacturer’s package insert for the HiPro Biotechnology HP-AFS Automatic Immunoassay System (HiPro Biotechnology HP-AFS/1-PLUS Automatic Immunoassay System (Hipro Biotechnology Co., Ltd., Shijiazhuang, China).): TG ≥ 1.69 mmol/L. TC, HDL-C, and LDL-C were not analyzed because of the analytical reliability concerns described in Section 2. Abnormal CRP was defined as >3.0 mg/L, consistent with the high cardiovascular-risk tertile established by the CDC/American Heart Association scientific statement on inflammatory markers [27]. Cardiometabolic sex-specific cut-off points for abnormal leptin were used, based on a large population-based reference study, namely >6.45 ng/mL for men and >23.75 ng/mL for women [28]. No established clinical or population reference range exists for serum ghrelin in young, non-obese adults; abnormal ghrelin was therefore defined using a sample-derived median split across the full sample, consistent with standard practice for hormones lacking a validated clinical threshold. This category should be interpreted as sample-relative rather than clinically diagnostic. Abnormal resting heart rate was defined as >80 bpm around the population mixture-analysis thresholds separating normal and elevated rates of resting heart rate [29]. Blood pressures were considered abnormal if either the systolic or diastolic pressure was ≥130/85 mmHg, in accordance with the blood-pressure criteria of the metabolic syndrome used in the Adult Treatment Panel III (ATP III) [30].

## 3. Results

### 3.1. Participant Characteristics and Mediterranean Diet Adherence

Of the 80 participants (44 males and 36 females; 80% aged < 25 years), 32 (40.0%) were classified as adherent and 48 (60.0%) as non-adherent. The majority were single (95.0%) and undergraduate students (83.8%); 42.5% were current smokers and 48.8% were non-smokers. Participant characteristics and their unadjusted distributions across MEDAS adherence groups are presented in Table 1. The crude distributions were broadly comparable between groups. These exploratory comparisons were used solely to characterize the sample and were not used to infer independent associations or to select covariates for the regression models.

### 3.2. Metabolic and Physiological Indicators

Adherent participants had significantly lower median TG concentrations (0.44 vs. 1.28 mmol/L; U = 388.00, *p* < 0.001) and lower FBG (87.00 vs. 91.00 mg/dL; U = 521.00, *p* = 0.015) compared with non-adherent participants. No significant between-group differences were observed for resting heart rate, SBP, or DBP (all *p* > 0.05; Table 2). TC, HDL-C, and LDL-C were not analyzed because of the analytical reliability concerns described in Section 2.

### 3.3. Inflammatory and Hormonal Biomarkers

Median CRP was significantly lower in adherent participants (1.10 vs. 1.75 mg/L; U = 517.00, *p* = 0.014). No statistically significant between-group difference in IL-6 was detected (U = 659.00, *p* = 0.227). However, interpretation was limited because 62.5% of measurements were below the assay detection limit, rendering this comparison underpowered by design. Bivariate comparisons did not reveal significant differences in leptin (U = 617.00, *p* = 0.138) or ghrelin (U = 729.00, *p* = 0.702; Table 2).

### 3.4. Anthropometric and Body Composition

Adherent participants had significantly lower BMI (21.10 vs. 24.45 kg/m^2^; U = 255.5, *p* < 0.001), BFM (14.15 vs. 20.15 kg; U = 413.0, *p* < 0.001), and PBF (23.55% vs. 26.65%; U = 536.0, *p* = 0.023). No significant between-group differences were found for FFM, TBW, SMM, or SMI (all *p* > 0.05; Table 2).

### 3.5. Behavioral Health Indicators and HRQoL

Exploratory unadjusted comparisons showed broadly similar distributions of sleep quality, physical activity level, and perceived recovery across MEDAS adherence groups (Table 3). These comparisons were descriptive and were not interpreted as evidence of independent associations. Poor sleep quality (PSQI > 5) was observed in 71.3% of the total sample, irrespective of adherence group. Adherent participants had a significantly higher median physical-oriented HRQoL score (83.33% vs. 72.22%; U = 515.5, *p* = 0.012), while mental-oriented HRQoL scores did not differ significantly between groups (*p* = 0.760; Table 2). No statistically significant between-group differences were observed in recorded energy, macronutrient, or micronutrient intakes (Supplementary Table S1).

### 3.6. Correlation Analysis

In the Spearman correlation matrix, BMI showed the strongest negative correlation with the continuous MEDAS score (rs = −0.577, *p* < 0.01), followed by CRP (rs = −0.287, *p* < 0.05), PBF (rs = −0.275, *p* < 0.05), TG (rs = −0.271, *p* < 0.05), and FBG (rs = −0.249, *p* < 0.05). Physical HRQoL was positively correlated with the MEDAS score (rs = 0.237, *p* < 0.05). The strongest inter-variable association was between leptin and PBF (rs = 0.831, *p* < 0.01). PSQI score, mental HRQoL, leptin, IL-6, and ghrelin showed no significant correlations with the MEDAS score (all *p* > 0.05; Table 4).

### 3.7. Logistic Regression

In exploratory models, MEDAS non-adherence was associated with higher odds of abnormal TG (adjusted OR = 9.17, 95% CI: 2.08–40.38, *p* = 0.003). In sensitivity models additionally adjusted for PBF, non-adherence remained associated with abnormal CRP (adjusted OR = 5.39, 95% CI: 1.34–21.61, *p* = 0.018), whereas the association with abnormal leptin was attenuated and was no longer statistically significant (adjusted OR = 3.47, 95% CI: 0.75–16.07, *p* = 0.111). The wide confidence intervals indicate limited precision, and these findings should be interpreted as exploratory, hypothesis-generating associations rather than as independent or causal effects. No significant adjusted associations were found for abnormal ghrelin, FBG, RHR, SBP, or DBP. The association between non-adherence and abnormal IL-6 could not be reliably evaluated, as 62.5% of IL-6 measurements fell below the assay detection limit, precluding meaningful group discrimination for this analyte. The DBP model showed suboptimal fit (Hosmer–Lemeshow *p* = 0.045) and should be interpreted with caution. These findings are exploratory given the sample size (Table 5).

## 4. Discussion

To our knowledge, this study is among the first in the Arab region to simultaneously examine Mediterranean diet adherence in relation to cardiometabolic, inflammatory, hormonal, body-composition, and behavioral-health indicators in physically active young adults, integrating objective biomarker assays with validated behavioral instruments. (1) MEDAS non-adherence was associated with higher odds of abnormal TG, while sensitivity analyses additionally adjusting for PBF showed that the CRP association remained significant but the leptin association was attenuated and no longer statistically significant; (2) adherent participants had lower BMI, BFM, and PBF, without corresponding differences in lean mass; and (3) 60% of physically active participants were non-adherent, indicating that physical activity participation did not necessarily coincide with high dietary quality. This association of MEDAS non-adherence with abnormal TG is of note. The association between MD adherence and lower triglyceride concentrations is biologically plausible and may partly reflect the characteristic intake of extra-virgin olive oil, fish, and nuts; extra-virgin olive oil and nuts provide monounsaturated fatty acids, whereas fish provides omega-3 polyunsaturated fatty acids, which are known to suppress hepatic VLDL synthesis and promote fatty acid oxidation [31]. In a meta-analysis of observational studies, increased MD adherence was linked to decreased TG levels and improved metabolic syndrome parameters [4]. The present findings extend this evidence to physically active young adults in Jordan, where meeting physical activity criteria did not preclude a less favorable triglyceride profile among participants with poor dietary adherence. Although the median values should not be interpreted diagnostically, the higher triglyceride concentrations among non-adherent participants indicate a comparatively less favorable cardiometabolic profile. TC, HDL-C, and LDL-C were not included in the present analysis because of analytical reliability concerns identified during data verification (see Section Strengths and Limitations). Recent prospective evidence indicates that higher plasma triglyceride concentrations in young adulthood are associated with an increased long-term risk of premature cardiovascular events [32].

The association between non-adherence and elevated CRP is consistent with the established anti-inflammatory properties of the MD, mediated through inhibition of nuclear factor-kappa B (NF-κB) signaling and diet-related modulation of gut-derived metabolites, including short-chain fatty acids [33,34]. A systematic review and meta-analysis of randomized controlled trials reported that MD-based interventions were associated with reductions in selected inflammatory biomarkers, including hs-CRP, IL-6, and IL-17, supporting the broader anti-inflammatory plausibility of the MD pattern [35]. This evidence should be interpreted cautiously in relation to the present CRP finding, as the meta-analysis did not show a significant pooled effect for CRP specifically. An important confounding consideration is that 42.5% of our sample were current smokers, with 55.9% of smokers in the non-adherent group. Since smoking independently elevates CRP through NF-κB activation and oxidative stress pathways, the observed association between non-adherence and elevated CRP may still partly reflect residual confounding by smoking and other unmeasured factors and therefore should not be interpreted as an independent dietary effect [36,37]. Because smoking was not included in the present model, the CRP estimate should be interpreted as potentially confounded and hypothesis-generating rather than as an independent dietary association. Larger studies with adequate event counts should allow more comprehensive simultaneous adjustment for smoking, adiposity, and other relevant confounders when evaluating inflammatory outcomes. The non-significant IL-6 result must be interpreted as analytically inconclusive rather than as evidence of a true null association. With 62.5% of IL-6 values below the assay LOD and substituted at LOD/2, between-group discrimination was severely restricted, and any group comparison for this analyte is underpowered by design. This is biologically coherent: resting serum IL-6 is typically very low in healthy, non-obese, physically active young adults, and habitual aerobic training further suppresses basal IL-6 concentrations [38]. A high-sensitivity immunoassay platform would be required to detect meaningful dietary effects on IL-6 in this population.

The association between MEDAS non-adherence and abnormal leptin was no longer statistically significant after additional adjustment for PBF (adjusted OR = 3.47, 95% CI: 0.75–16.07, *p* = 0.111). This finding is consistent with the strong correlation between leptin and PBF (r_s_ = 0.831, *p* < 0.01) and indicates that the leptin association was sensitive to adjustment for adiposity. In this cross-sectional design, adiposity may act as either a confounder or an intermediate factor linking dietary adherence to leptin concentrations; therefore, causal ordering cannot be established. Sureda et al. (2018) reported that higher MD adherence was linked to lower leptin in females, consistent with an adiposity-mediated pathway [39]. Leptin additionally functions as a pro-inflammatory adipokine positively correlated with CRP [40], and the co-occurrence of elevated leptin and CRP among non-adherent participants is consistent with the broader pattern of adiposity-linked cardiometabolic dysregulation documented in the literature. No association was detected between MD adherence and fasting ghrelin. Ghrelin concentrations are influenced by recent energy balance, meal timing, exercise timing, and substantial intra-individual variability; therefore, a single fasting measurement may have had limited sensitivity to detect dietary-pattern differences [7]. Under controlled fasting conditions, leptin administration did not alter circulating ghrelin concentrations, supporting the physiological distinction between these two hormonal pathways [41]. Accordingly, the present result should be interpreted as a null finding within this study rather than evidence that no diet–ghrelin relationship exists.

The significantly lower BMI, BFM, and PBF in adherent participants, without a corresponding difference in FFM, TBW, SMM, or SMI, suggests that MD adherence in this population is associated with a lower adiposity profile rather than differences in lean mass. The difference in BMI (21.10 vs. 24.45 kg/m^2^) positions adherent participants within the normal-weight range while non-adherent participants approach the overweight threshold, reflecting a clinically meaningful dietary quality-adiposity gradient even within a physically active sample. No significant differences were detected in fat-free mass, skeletal muscle mass, or skeletal muscle index. These findings suggest that the observed body-composition differences were primarily related to adiposity rather than lean mass. Intervention studies combining Mediterranean diet adherence with structured exercise have reported improvements in anthropometric and cardiometabolic indicators, although direct comparison with the present cross-sectional lean-mass findings should be made cautiously [42]. The non-significant TBW difference is physiologically plausible, as TBW is closely related to fat-free mass rather than dietary pattern quality per se [43,44]. The three-day dietary records showed no significant between-group differences in energy, nutrient, or other recorded dietary components (Supplementary Table S1). This may reflect the relatively short recording period, within-person dietary variability, and the distinction between food-pattern adherence captured by MEDAS and absolute nutrient intake captured by dietary records. Nevertheless, the absence of convergent differences in dietary records should be considered when interpreting the adherence-group comparisons. MD adherence was associated with better physical-oriented HRQoL but not mental-oriented HRQoL. A recent systematic review found that greater Mediterranean diet adherence was generally associated with better HRQoL, although findings varied across populations and physical and mental health domains [9]. The physical HRQoL association seems multi-determined as the correlation matrix revealed that physical HRQoL was inversely associated with TG (rs = −0.267), BMI (rs = −0.259) and the PSQI score (rs = −0.229), indicating that the connection between dietary quality and physical functioning may operate in part through metabolic and adiposity-related pathways. The non-significant mental HRQoL finding could be due to the fact that other psychosocial factors that are not measured in the current study may have influenced this finding, such as perceived stress or contextual life demands. In future studies, direct assessment of stress and psychological burden should be incorporated in the study of diet–HRQoL relationships in young adults. However, it is worth emphasizing that poor sleep quality was highly prevalent, affecting 71.3% of the total sample. No significant association was observed between MEDAS adherence and PSQI score, possibly because poor sleep was highly prevalent in both groups, thereby limiting between-group variability. The correlation matrix showed that PSQI score was positively correlated with TG (rs = 0.261) and inversely correlated with mental-oriented HRQoL (rs = −0.258). These findings identify sleep quality as an important correlate, but do not establish that it acted as a confounder in the observed associations. The similarly high physical-activity levels in both groups may also have reduced the ability to detect dietary differences in sleep outcomes [45]. Although modest positive associations between physical activity and Mediterranean diet adherence have been reported among Croatian fitness-center users and Portuguese school teachers, being physically active does not necessarily ensure high dietary quality [46,47]. Although physical activity is independently valuable for metabolic health, differences in TG and CRP remained evident across MEDAS adherence groups, whereas the association with abnormal leptin was no longer statistically significant after adjustment for PBF. These findings suggest that dietary quality and physical activity should not be treated as interchangeable health behaviors, a distinction that warrants further evaluation in future public-health and university-based research in Jordan.

### Strengths and Limitations

A major strength of this study was the integration of metabolic, inflammatory, and appetite-related biomarkers with multifrequency bioelectrical-impedance measurements and validated behavioral-health instruments in a clearly defined physically active Jordanian population. Nevertheless, the cross-sectional design precluded causal inference and assessment of directionality. The purposively selected sample from fitness centers and university campuses limits generalizability to the wider Jordanian population. The modest sample size and sparse outcome events necessitated parsimonious regression models; therefore, the adjusted estimates, including the additional PBF-adjusted sensitivity models for CRP and leptin, should be interpreted as exploratory and hypothesis-generating rather than as independent effects. Residual confounding by smoking, exercise type and volume, and other lifestyle factors cannot be excluded; moreover, adiposity may act as either a confounder or an intermediate factor in selected biomarker associations. The large number of exploratory comparisons increases the possibility of type I error. Because the analyses were hypothesis-generating rather than confirmatory, no formal multiplicity correction was applied; nominal *p*-values should therefore be interpreted cautiously and require replication. The continuous MEDAS correlations and continuous group comparisons complemented the categorized MEDAS and abnormal-outcome analyses, although categorization may have reduced information and statistical power. The study was not designed or powered for sex-stratified or MEDAS-by-sex interaction analyses; gender was therefore included as an adjustment variable, and potential effect modification should be evaluated in larger samples. The MEDAS was validated in Moroccan Arabic rather than specifically in Jordan. Although validated instruments were used, the self-reported MEDAS, IPAQ-SF, and three-day dietary records remain susceptible to recall and social-desirability bias. Accordingly, findings derived from self-reported measures were interpreted cautiously and alongside the objective laboratory and body-composition assessments. Interpretation of IL-6 was limited because 62.5% of measurements were below the assay limit of detection. During verification of the lipid panel, HDL-C showed an implausible distribution (41 of 77 valid measurements at exactly 3.55 mmol/L, despite a validated linearity range of 0.10–3.90 mmol/L and no differential sample handling across participants), with three further values below the assay’s lower analytical limit and no identifiable technical explanation; because total cholesterol was measured on the same platform in the same analytical run and LDL-C was calculated from total cholesterol, HDL-C, and triglycerides, the reliability of all three analytes could not be established, and they were therefore excluded from analysis. The three-day dietary records may not fully represent habitual intake.

## 5. Conclusions

In this exploratory cross-sectional sample of 80 physically active young adults purposively recruited in Amman, Mediterranean diet non-adherence was associated with higher triglyceride concentrations, greater adiposity, lower physical-oriented HRQoL, and higher odds of abnormal TG and CRP; however, the association with abnormal leptin was not statistically significant after additional adjustment for PBF. These findings should not be generalized to the wider Jordanian young-adult population and require confirmation in larger, representative samples. The observed associations are hypothesis-generating rather than confirmatory, and prospective studies with more comprehensive confounder adjustment are needed.

## Figures and Tables

**Table 1:** Participant Characteristics by Mediterranean Diet Adherence Group (N = 80).

Variable	Category	All Participants n (%)	Adherent (n = 32), n (%)	Non-Adherent (n = 48), n (%)	χ^2^	*p*-Value	φ/V
Age *		21 (20, 24)	21 (20, 24.75)	21 (20, 23.75)	735.0	0.743 **	0.04
Gender	Male	44 (55.0)	19 (43.2)	25 (56.8)	0.41	0.521	0.07
	Female	36 (45.0)	13 (36.1)	23 (63.9)			
Age (years)	<25	64 (80.0)	28 (43.8)	36 (56.3)	1.88	0.171	0.15
	≥25	16 (20.0)	4 (25.0)	12 (75.0)			
Marital status	Single	76 (95.0)	30 (39.5)	46 (60.5)	0.18	0.675	0.05
	Married	4 (5.0)	2 (50.0)	2 (50.0)			
Education level	Undergraduate	67 (83.8)	26 (38.8)	41 (61.2)	0.25	0.621	0.06
	Postgraduate	13 (16.3)	6 (46.2)	7 (53.8)			
Smoking status	Current smoker	34 (42.5)	15 (44.1)	19 (55.9)	2.19	0.335	0.17
	Former smoker	7 (8.8)	1 (14.3)	6 (85.7)			
	Non-smoker	39 (48.8)	16 (41.0)	23 (59.0)			
Income level (JD)	<300	39 (48.7)	14 (35.9)	25 (64.1)	1.14	0.566	0.12
	300–500	20 (25.0)	10 (50.0)	10 (50.0)			
	>500	21 (26.3)	8 (38.1)	13 (61.9)			

Note. Data are presented as n (%), except age, which is presented as median (IQR). * Age values are presented as median (IQR). ** Wilcoxon–Mann–Whitney U test. For continuous age, 735.0 represents the Mann–Whitney U statistic and 0.04 represents the corresponding effect size. Adherence was defined as a MEDAS score ≥ 8. JD = Jordanian dinar. Percentages in the adherence-group columns are row percentages, whereas percentages in the total-sample column are column percentages. Between-group comparisons were performed using Pearson’s chi-square test or Fisher’s exact test, as appropriate. These analyses were unadjusted and exploratory and were used only to characterize the distribution of participant characteristics across MEDAS adherence groups; they were not used to establish independent associations or to select covariates for the multivariable models. Effect sizes are reported as Phi (φ) for 2 × 2 tables and Cramér’s V for variables with more than two categories.

**Table 2:** Clinical, Biomarker, Anthropometric, Body-Composition, and HRQoL Indicators by Mediterranean Diet Adherence (N = 80).

Domain	Variable/Outcome	Adherent (n = 32)	Non-Adherent (n = 48)	Mann–Whitney U	*p*-Value
Metabolic indicators	FBG (mg/dL)	87.00 (81.25–91.75)	91.00 (86.50–95.00)	521.00	0.015
	Triglycerides (mmol/L)	0.44 (0.35–1.11)	1.28 (0.80–2.89)	388.00	<0.001
Physiological indicators	Heart rate (bpm)	77 (65–90.75)	76.5 (69.25–89.0)	763.500	0.965
	SBP (mmHg)	113.5 (104.25–128.0)	119.50 (107.25–130.0)	838.500	0.488
	DBP (mmHg)	81 (75.25–86.75)	81 (77.0–85.75)	767.500	0.966
Inflammatory biomarkers	CRP (mg/L) ^a^	1.10 (0.73–3.10)	1.75 (1.20–3.63)	517.00	0.014
	IL-6 (pg/mL) ^b^	1.41 (1.41–5.87)	1.41 (1.41–5.25)	659.00	0.227
Appetite-related hormones	Ghrelin (pg/mL)	45.92 (28.43–60.53)	38.38 (26.66–56.43)	729.00	0.702
	Leptin (ng/mL)	5.66 (4.01–11.50)	7.53 (3.86–15.08)	617.00	0.138
Anthropometric and body- composition indicators ^c^	BMI (kg/m^2^)	21.10 (19.45–22.58)	24.45 (23.55–26.88)	255.5	<0.001
	BFM (kg)	14.15 (10.45–18.98)	20.15 (15.53–25.17)	413.0	<0.001
	PBF (%)	23.55 (14.10–28.40)	26.65 (19.80–37.08)	536.0	0.023
	TBW (L)	36.50 (28.83–45.03)	38.40 (31.88–45.75)	690.0	0.444
	FFM (kg)	49.80 (39.38–61.68)	52.45 (43.70–62.70)	689.0	0.438
	SMM (kg)	26.55 (23.85–35.53)	28.20 (20.95–34.92)	686.5	0.423
	SMI (kg/m^2^)	7.30 (6.52–8.07)	7.10 (5.92–8.07)	618.0	0.140
Health-related quality of life	Physical-oriented HRQoL score (%)	83.33 (72.22–93.06)	72.22 (61.11–88.89)	515.5	0.012
	Mental-oriented HRQoL score (%)	68.00 (56.00–79.00)	68.00 (60.00–76.00)	737.0	0.760

Note. Values are presented as median (Q1–Q3). IQR = Interquartile Range. Mann–Whitney U = Mann–Whitney U test. MEDAS = Mediterranean Diet Adherence Screener. Adherence was defined as MEDAS score ≥ 8. *p*-values are two-tailed. A nominal *p*-value < 0.05 was considered statistically significant. FBG = Fasting Blood Glucose. SBP = Systolic Blood Pressure. DBP = Diastolic Blood Pressure. bpm = beats per minute. CRP = C-Reactive Protein. IL-6 = Interleukin-6. BMI = Body Mass Index. BFM = Body Fat Mass. PBF = Percent Body Fat. TBW = Total Body Water. FFM = Fat-Free Mass. SMM = Skeletal Muscle Mass. SMI = Skeletal Muscle Index. HRQoL = Health-Related Quality of Life. Physical- and mental-oriented HRQoL were assessed using the SF-12 Health Survey and expressed as percentage scores for physical and mental components. Total cholesterol, HDL-C, and LDL-C are not reported in this table because of analytical reliability concerns identified during data verification (see Section 2 and Section Strengths and Limitations). ^a^ CRP values exceeding the upper limit of quantification (10.0 mg/L) were top-coded at 10.0 mg/L. ^b^ IL-6 values below the limit of detection (LOD) were substituted at LOD/2; 62.5% of IL-6 values fell below the LOD, which constitutes a methodological limitation and renders group comparisons for this variable exploratory only. ^c^ Body composition was assessed using InBody 970 multifrequency bioelectrical impedance analysis. Between-group tests represent unadjusted exploratory comparisons. Because no formal multiplicity correction was applied, *p*-values should be interpreted cautiously and not as confirmatory evidence.

**Table 3:** Behavioral Health Indicators and Their Associations with Mediterranean Diet Adherence (N = 80).

Variable	Adherent (n = 32)	Non-Adherent (n = 48)	χ^2^	*p*-Value	φ/Cramér’s V
**Sleep quality**					
Poor	24 (42.1%)	33 (57.9%)	0.37	0.545	φ = 0.07
Good	8 (34.8%)	15 (65.2%)			
**Physical activity level**					
Moderate active	15 (37.5%)	25 (62.5%)	0.21	0.648	φ = 0.05
High active	17 (42.5%)	23 (57.5%)			
**Perceived recovery scale**					
Poor	2 (18.2%)	9 (81.8%)	2.79	0.248	Cramér’s V = 0.19
Moderate	24 (42.1%)	33 (57.9%)			
Excellent	6 (50.0%)	6 (50.0%)			

Note. Data are presented as n (row %). Adherence was defined as MEDAS score ≥ 8. Sleep quality was classified using the global Pittsburgh Sleep Quality Index (PSQI) score. Physical activity level was categorized using the International Physical Activity Questionnaire (IPAQ). Perceived recovery was classified using the Perceived Recovery Status (PRS) scale. Group comparisons were performed using Pearson’s chi-square test or Fisher’s exact test as appropriate. Effect sizes are reported as Phi (φ) for 2 × 2 tables and Cramér’s V for variables with more than two categories. These analyses were unadjusted and exploratory and were used only to describe the distributions of behavioral indicators across adherence groups. They were not interpreted as independent associations.

**Table 4:** Spearman Correlations Between MEDAS Score and Selected Health Outcomes (N = 80).

Variable	1	2	3	4	5	6	7	8	9	10
1. MEDAS score	—									
2. FBG (mg/dL)	−0.249 *	—								
3. Triglycerides (mmol/L)	−0.271 *	0.121	—							
4. CRP (mg/L)	−0.287 *	0.045	−0.090	—						
5. Leptin (ng/mL)	−0.131	−0.120	0.335 **	0.129	—					
6. Fat Mass %	−0.275 *	−0.176	0.204	0.159	0.831 **	—				
7. BMI (kg/m^2^)	−0.577 **	0.292 **	0.329 **	0.167	0.302 **	0.432 **	—			
8. PSQI score	0.005	−0.179	0.261 *	−0.107	0.047	0.049	0.115	—		
9. Physical HRQoL (%)	0.237 *	−0.020	−0.267 *	−0.017	−0.177	−0.216	−0.259 *	−0.229 *	—	
10. Mental HRQoL (%)	0.026	0.005	−0.148	0.155	0.049	−0.038	−0.133	−0.258 *	0.426 **	—

Note. N = 80. MEDAS = Mediterranean Diet Adherence Screener. Spearman’s rho coefficients are reported. FBG = Fasting Blood Glucose. CRP = C-Reactive Protein. BMI = Body Mass Index. PSQI = Pittsburgh Sleep Quality Index. HRQoL = Health-Related Quality of Life. * *p* < 0.05, two-tailed. ** *p* < 0.01, two-tailed. Correlations were exploratory and unadjusted, and *p*-values were not corrected for multiple testing. The continuous MEDAS analysis was included to complement the categorical adherence comparisons and to preserve information contained in the full 0–14 score.

**Table 5:** Exploratory Adjusted Associations Between Mediterranean Diet Non-Adherence and Abnormal Clinical and Laboratory Outcomes (N = 80).

Outcome	Adjusted OR	95% CI	*p*-Value	Model χ^2^ (df)	Nagelkerke R^2^	Hosmer–Lemeshow *p*-Value
Abnormal CRP	5.385	1.343–21.605	0.018	15.776 (4)	0.269	0.460
Abnormal TG	9.172	2.083–40.379	0.003	11.723 (3)	0.210	0.797
Abnormal IL-6	1.450	0.473–4.440	0.516	2.764 (3)	0.050	0.694
Abnormal Ghrelin	0.497	0.151–1.632	0.249	6.605 (3)	0.117	0.084
Abnormal Leptin	3.473	0.750–16.073	0.111	23.954 (4)	0.378	0.927
Abnormal RHR	1.410	0.317–6.276	0.652	3.230 (3)	0.075	0.527
Abnormal FBG	1.006	0.207–4.889	0.994	1.427 (3)	0.035	0.571
Abnormal SBP	0.700	0.260–1.884	0.480	1.130 (3)	0.019	0.452
Abnormal DBP ^†^	1.151	0.433–3.057	0.778	0.579 (3)	0.010	0.045

Note. CRP = C-reactive protein; TG = triglycerides; IL-6 = interleukin-6; RHR = resting heart rate; FBG = fasting blood glucose; SBP = systolic blood pressure; DBP = diastolic blood pressure; OR = odds ratio; CI = confidence interval; df = degrees of freedom; PBF = percent body fat. For outcomes other than CRP and leptin, odds ratios were obtained from hierarchical models adjusted for age and gender, with MEDAS adherence added in Block 2. For CRP and leptin, the estimates shown are from sensitivity models additionally adjusted for PBF to assess the influence of adiposity on these associations. Given the modest sample size and sparse outcome events, estimates are exploratory and should not be interpreted as independent or causal effects. Abnormal LDL-C is not reported because LDL-C was not analyzed (see Section 2 and Section Strengths and Limitations). ^†^ The DBP model showed inadequate fit according to the Hosmer–Lemeshow test (*p* = 0.045) and should be interpreted cautiously.

## Data Availability

The data supporting the findings of this study are available from the corresponding author upon reasonable request.

## Supplementary Materials

The following supporting information can be downloaded at: https://www.mdpi.com/article/10.3390/healthcare14193345/s1, Table S1: Mean Daily Energy, Macronutrient, and Micronutrient Intakes by Mediterranean Diet Adherence (N = 80).

## References

[1] Lopes S., Fontes T., Menezes R., Rodrigues L., Ferreira-Pêgo C. (2023). Mediterranean Diet Adherence and Its Relationship to Metabolic Markers and Body Composition in Portuguese University Students. Nutrients.

[2] Estruch R., Ros E., Salas-Salvadó J., Covas M.-I., Corella D., Arós F., Gómez-Gracia E., Ruiz-Gutiérrez V., Fiol M., Lapetra J. (2018). Primary Prevention of Cardiovascular Disease with a Mediterranean Diet Supplemented with Extra-Virgin Olive Oil or Nuts. N. Engl. J. Med..

[3] Calella P., Gallè F., Di Onofrio V., Cerullo G., Liguori G., Valerio G. (2022). Adherence to Mediterranean Diet in Athletes: A Narrative Review. Sport Sci. Health.

[4] Bakaloudi D.R., Chrysoula L., Kotzakioulafi E., Theodoridis X., Chourdakis M. (2021). Impact of the Level of Adherence to Mediterranean Diet on the Parameters of Metabolic Syndrome: A Systematic Review and Meta-Analysis of Observational Studies. Nutrients.

[5] Koelman L., Egea Rodrigues C., Aleksandrova K. (2022). Effects of Dietary Patterns on Biomarkers of Inflammation and Immune Responses: A Systematic Review and Meta-Analysis of Randomized Controlled Trials. Adv. Nutr..

[6] Manzano-Carrasco S., Felipe J.L., Sanchez-Sanchez J., Hernandez-Martin A., Gallardo L., Garcia-Unanue J. (2020). Physical Fitness, Body Composition, and Adherence to the Mediterranean Diet in Young Football Players: Influence of the 20 MSRT Score and Maturational Stage. Int. J. Environ. Res. Public Health.

[7] Skoracka K., Hryhorowicz S., Schulz P., Zawada A., Ratajczak-Pawłowska A.E., Rychter A.M., Słomski R., Dobrowolska A., Krela-Kaźmierczak I. (2025). The Role of Leptin and Ghrelin in the Regulation of Appetite in Obesity. Peptides.

[8] Godos J., Grosso G., Castellano S., Galvano F., Caraci F., Ferri R. (2021). Association between Diet and Sleep Quality: A Systematic Review. Sleep Med. Rev..

[9] Godos J., Guglielmetti M., Ferraris C., Frias-Toral E., Domínguez Azpíroz I., Lipari V., Di Mauro A., Furnari F., Castellano S., Galvano F. (2025). Mediterranean Diet and Quality of Life in Adults: A Systematic Review. Nutrients.

[10] Basha H., Shalash A., Wahdan Y., Abu-Rmeileh N.M.E. (2025). Shifts in Food Consumption Patterns in the Levant: A Systematic Review of the Last Six Decades. Int. J. Behav. Nutr. Phys. Act..

[11] Al-Awwad N.J., Ayoub J., Barham R., Sarhan W., Al-Holy M., Abughoush M., Al-Hourani H., Olaimat A., Al-Jawaldeh A. (2021). Review of the Nutrition Situation in Jordan: Trends and Way Forward. Nutrients.

[12] Al Hourani H., Alkhatib B., Al-Shami I., Olaimat A.N., Al-Holy M., Al-Awwad N.J., Abughoush M., Saleh N.A., AlHalaika D., Alboqai O. (2023). Energy and Macronutrient Intakes in Jordan: A Population Study. Sci. Rep..

[13] Alkhawaldeh A., Abdalrahim A., ALBashtawy M., Ayed A., Al Omari O., ALBashtawy S., Suliman M., Oweidat I.A., Khatatbeh H., Alkhawaldeh H. (2024). University Students’ Physical Activity: Perceived Barriers and Benefits to Physical Activity and Its Contributing Factors. SAGE Open Nurs..

[14] von Elm E., Altman D.G., Egger M., Pocock S.J., Gøtzsche P.C., Vandenbroucke J.P. (2007). The Strengthening the Reporting of Observational Studies in Epidemiology (STROBE) Statement: Guidelines for Reporting Observational Studies. Lancet.

[15] WHO (2020). Guidelines on Physical Activity and Sedentary Behaviour.

[16] Labib B., Khader Y., Abu Khudair S., Al Nsour M., Tanaka E. (2024). The Prevalence of Behavioral and Emotional Problems and Their Associated Factors Among Children and Adolescents in Jordan: Findings from a National School-Based Survey. Pediatr. Rep..

[17] Faul F., Erdfelder E., Lang A.-G., Buchner A. (2007). G*Power 3: A Flexible Statistical Power Analysis Program for the Social, Behavioral, and Biomedical Sciences. Behav. Res. Methods.

[18] Sammoud K., Mahdi Z., Benzaida K., Elrhaffouli Y., Yamlahi M., Gourinda A., Charif F., Bousgheiri F., Elbouri H., Adil N. (2023). Cross-Cultural Adaptation of Mediterranean Diet Adherence Screener (MEDAS) Into Moroccan Arabic to Measure the Degree of Mediterranean Diet Adherence. Cureus.

[19] Lubin J.H., Colt J.S., Camann D., Davis S., Cerhan J.R., Severson R.K., Bernstein L., Hartge P. (2004). Epidemiologic Evaluation of Measurement Data in the Presence of Detection Limits. Environ. Health Perspect..

[20] Hornung R.W., Reed L.D. (1990). Estimation of Average Concentration in the Presence of Nondetectable Values. Appl. Occup. Environ. Hyg..

[21] Gabay C., Kushner I. (1999). Acute-Phase Proteins and Other Systemic Responses to Inflammation. N. Engl. J. Med..

[22] ElSayed N.A., Aleppo G., Aroda V.R., Bannuru R.R., Brown F.M., Bruemmer D., Collins B.S., Hilliard M.E., Isaacs D., Johnson E.L. (2023). 6. Glycemic Targets: Standards of Care in Diabetes—2023. Diabetes Care.

[23] Craig C.L., Marshall A.L., Sjöström M.M., Bauman A.E., Booth M.L., Ainsworth B.E., Pratt M., Ekelund U., Yngve A., Sallis J.F. (2003). International Physical Activity Questionnaire: 12-Country Reliability and Validity. Med. Sci. Sports Exerc..

[24] Suleiman K.H., Yates B.C., Berger A.M., Pozehl B., Meza J. (2010). Translating the Pittsburgh Sleep Quality Index into Arabic. West. J. Nurs. Res..

[25] Laurent C.M., Green J.M., Bishop P.A., Sjökvist J., Schumacker R.E., Richardson M.T., Curtner-Smith M. (2011). A Practical Approach to Monitoring Recovery: Development of a Perceived Recovery Status Scale. J. Strength Cond. Res..

[26] Haddad C., Sacre H., Obeid S., Salameh P., Hallit S. (2021). Validation of the Arabic Version of the “12-Item Short-Form Health Survey” (SF-12) in a Sample of Lebanese Adults. Arch. Public Health.

[27] Pearson T.A., Mensah G.A., Alexander R.W., Anderson J.L., Cannon R.O., Criqui M., Fadl Y.Y., Fortmann S.P., Hong Y., Myers G.L. (2003). Markers of Inflammation and Cardiovascular Disease. Circulation.

[28] Gijón-Conde T., Graciani A., Guallar-Castillón P., Aguilera M.T., Rodríguez-Artalejo F., Banegas J.R. (2015). Leptin Reference Values and Cutoffs for Identifying Cardiometabolic Abnormalities in the Spanish Population. Rev. Española Cardiol..

[29] Palatini P. (1999). Need for a Revision of the Normal Limits of Resting Heart Rate. Hypertension.

[30] Cleeman J.I. (2001). Expert Panel on Detection Evaluation and Treatment of High Blood Cholesterol in Adults Executive Summary of the Third Report of the National Cholesterol Education Program (NCEP) Expert Panel on Detection, Evaluation, and Treatment of High Blood Cholesterol in Adults (Adult Treatment Panel III). JAMA J. Am. Med. Assoc..

[31] Calder P.C. (2022). Omega-3 Fatty Acids and Metabolic Partitioning of Fatty Acids within the Liver in the Context of Nonalcoholic Fatty Liver Disease. Curr. Opin. Clin. Nutr. Metab. Care.

[32] Aaseth E., Halvorsen S., Helseth R., Gravning J. (2025). Remnant Cholesterol, Plasma Triglycerides, and Risk of Cardiovascular Disease Events in Young Adults: A Prospective Cohort Study. Eur. J. Prev. Cardiol..

[33] Fedullo A.L., Schiattarella A., Morlando M., Raguzzini A., Toti E., De Franciscis P., Peluso I. (2021). Mediterranean Diet for the Prevention of Gestational Diabetes in the Covid-19 Era: Implications of Il-6 In Diabesity. Int. J. Mol. Sci..

[34] Florkowski M., Abiona E., Frank K.M., Brichacek A.L. (2024). Obesity-Associated Inflammation Countered by a Mediterranean Diet: The Role of Gut-Derived Metabolites. Front. Nutr..

[35] Keshani M., Rafiee S., Heidari H., Rouhani M.H., Sharma M., Bagherniya M. (2025). Mediterranean Diet Reduces Inflammation in Adults: A Systematic Review and Meta-Analysis of Randomized Controlled Trials. Nutr. Rev..

[36] Addissouky T.A., El Sayed I.E.T., Ali M.M.A., Wang Y., El Baz A., Elarabany N., Khalil A.A. (2024). Oxidative Stress and Inflammation: Elucidating Mechanisms of Smoking-Attributable Pathology for Therapeutic Targeting. Bull. Natl. Res. Cent..

[37] Koh D.-H., Choi S., Park J.-H., Lee S.-G., Kim H.-C., Kim I., Han S.-S., Park D.-U. (2024). Assessing the Association between Cigarette Smoking and Blood C-Reactive Protein Levels Using Restructured Cohort Data. Prev. Med..

[38] Said E.A., Al-Reesi I., Al-Shizawi N., Jaju S., Al-Balushi M.S., Koh C.Y., Al-Jabri A.A., Jeyaseelan L. (2021). Defining IL-6 Levels in Healthy Individuals: A Meta-analysis. J. Med. Virol..

[39] Sureda A., del Mar Bibiloni M., Julibert A., Bouzas C., Argelich E., Llompart I., Pons A., Tur J.A. (2018). Adherence to the Mediterranean Diet and Inflammatory Markers. Nutrients.

[40] Graßmann S., Wirsching J., Eichelmann F., Aleksandrova K. (2017). Association Between Peripheral Adipokines and Inflammation Markers: A Systematic Review and Meta-Analysis. Obesity.

[41] Chan J.L., Bullen J., Lee J.H., Yiannakouris N., Mantzoros C.S. (2004). Ghrelin Levels Are Not Regulated by Recombinant Leptin Administration and/or Three Days of Fasting in Healthy Subjects. J. Clin. Endocrinol. Metab..

[42] Prieto-González P., Yagin F.H., Alghannam A.F., Canli U. (2025). Effects of a Mediterranean Diet and Structured Exercise Intervention on Selected Anthropometric, Cardiovascular, and Metabolic Variables in Physically Inactive Adults: A Randomized Controlled Trial. Front. Nutr..

[43] Kyle U. (2004). Bioelectrical Impedance Analysis? Part I: Review of Principles and Methods. Clin. Nutr..

[44] Liu S., Yang Y., Song J., Ma L., Wang Y., Mei Q., Jiang W. (2022). Total Body Water/Fat-Free Mass Ratio as a Valuable Predictive Parameter for Mortality in Maintenance Hemodialysis Patients. Medicine.

[45] Mantzorou M., Mentzelou M., Vasios G.K., Kontogiorgis C., Antasouras G., Vadikolias K., Psara E., Vorvolakos T., Poulios E., Serdari A. (2023). Mediterranean Diet Adherence Is Associated with Favorable Health-Related Quality of Life, Physical Activity, and Sleep Quality in a Community-Dwelling Greek Older Population. Antioxidants.

[46] Martinovic D., Tokic D., Martinovic L., Kumric M., Vilovic M., Rusic D., Vrdoljak J., Males I., Ticinovic Kurir T., Lupi-Ferandin S. (2021). Adherence to the Mediterranean Diet and Its Association with the Level of Physical Activity in Fitness Center Users: Croatian-Based Study. Nutrients.

[47] Esgalhado M., Raposo A., Albaridi N.A., Alslamah T., Alqarawi N., Oliveira L. (2025). Mediterranean Diet, Sleep Quality, Perceived Stress, and Physical Activity: A Pilot Study Among School Teachers. Nutrients.

