# Supplementary material for: Mediterranean Diet Adherence and Cardiometabolic, Inflammatory, Hormonal, Body Composition, and Behavioral Health Profiles in Physically Active Young Adults: An Analytical Cross-Sectional Study
Source: Healthcare (Basel). 2026 Oct 8;14(19):3345. doi: 10.3390/healthcare14193345 (PMC13665572; doi:10.3390/healthcare14193345)
Supplement: Supplementary file 1 [file healthcare-14-03345-s001.zip › healthcare-4491720-supplementary.pdf]

Supplementary Table S1. Mean Daily Energy, Macronutrient, and Micronutrient Intakes by Mediterranean Diet Adherence (N = 80)

Variable	Adherent (n=32)	Non-Adherent (n=48)	Mann-Whitney U	p-value
Weight (g)	1569.11 [1151.11, 1876.12]	1489.88 [1242.11, 1808.38]	733	0.731
Energy (kcal)	2005.09 [1410.39, 2892.57]	1926.57 [1509.42, 2468.20]	684	0.409
Fat calories (kcal)	759.00 [558.41, 1102.75]	671.26 [517.31, 1078.11]	670	0.336
Saturated fat calories (kcal)	191.58 [141.70, 336.17]	188.49 [127.37, 250.85]	635	0.191
Protein (g)	88.95 [67.58, 112.86]	85.94 [63.11, 124.54]	737	0.761
Carbohydrate (g)	224.86 [137.26, 324.73]	209.72 [151.96, 274.23]	682	0.398
Total fiber (g)	15.76 [7.98, 23.34]	13.93 [11.48, 19.74]	715	0.603
Total soluble fiber (g)	0.12 [0.00, 0.55]	0.13 [0.00, 0.55]	711	0.565
Fiber 16 (g)	9.10 [5.27, 13.03]	8.27 [5.28, 10.70]	729	0.702
Soluble fiber 16 (g)	0.05 [0.00, 0.39]	0.00 [0.00, 0.31]	663.5	0.273
Sugar (g)	56.67 [35.09, 83.07]	56.06 [38.14, 69.90]	696	0.480
Fat (g)	84.43 [62.23, 122.72]	74.60 [57.65, 119.83]	668.5	0.328
Saturated fat (g)	21.29 [15.75, 48.31]	22.05 [14.15, 29.00]	627	0.166
Water (g)	372.47 [207.80, 515.78]	318.69 [177.83, 455.12]	668	0.326
Carotene (mcg)	35.65 [10.61, 98.21]	21.68 [8.81, 51.99]	649.5	0.245
Vitamin B1 (mg)	1.10 [0.60, 1.59]	0.98 [0.57, 1.53]	678.5	0.379
Vitamin B2 (mg)	1.23 [0.77, 1.73]	1.32 [0.91, 1.85]	713	0.589
Vitamin B3-NE (mg)	22.33 [11.09, 39.06]	19.61 [8.96, 31.52]	668	0.326
Vitamin B6 (mg)	1.10 [0.50, 1.94]	0.90 [0.54, 1.74]	721	0.757
Vitamin B12 (mcg)	1.66 [0.99, 3.90]	1.43 [0.56, 3.53]	689	0.438
Vitamin C (mg)	31.20 [8.75, 92.23]	43.45 [8.68, 81.72]	735	0.746
Vitamin D (mcg)	1.32 [0.16, 1.82]	0.83 [0.02, 3.50]	767.5	0.996
Vitamin E (mg)	3.59 [2.53, 10.12]	5.36 [2.64, 9.66]	680.5	0.475
Folate (mcg)	259.45 [138.93, 387.03]	233.21 [125.00, 341.69]	677	0.371
Folate DFE (mcg DFE)	259.85 [143.13, 414.20]	241.38 [125.99, 365.54]	680	0.472
Calcium (mg)	575.11 [386.60, 787.41]	549.48 [336.05, 823.36]	724	0.666
Iron (mg)	11.19 [7.01, 15.77]	10.05 [6.98, 14.20]	677	0.371
Magnesium (mg)	127.62 [82.97, 192.39]	121.43 [74.24, 181.17]	707	0.549
Manganese (mg)	0.85 [0.34, 1.60]	0.55 [0.25, 1.24]	637	0.198
Phosphorus (mg)	703.39 [436.65, 1084.87]	646.08 [311.69, 900.06]	658	0.280
Potassium (mg)	1626.26 [1160.56, 2071.14]	1593.39 [1212.28, 2201.42]	757	0.914
Selenium (mcg)	61.70 [30.18, 100.62]	53.89 [32.16, 86.57]	691	0.542
Sodium (mg)	3799.04 [2167.00, 4874.26]	3036.41 [2086.13, 3945.37]	626	0.163
Zinc (mg)	4.68 [3.64, 7.24]	3.37 [2.38, 6.50]	591	0.082

Values are presented as median [Q1, Q3] of participants' mean daily intakes. Mean daily intake was calculated for each participant by averaging the three-day dietary record, which included two weekdays and one weekend day. Nutrient intake was analyzed using ESHA Food Processor, version 8.6.
